# Signatures of local adaptation to current and future climate in phenology-related genes in natural populations of *Quercus robur*

**DOI:** 10.1186/s12864-023-09897-y

**Published:** 2024-01-19

**Authors:** Joanna Meger, Bartosz Ulaszewski, Daniel J. Chmura, Jarosław Burczyk

**Affiliations:** 1 Department of Genetics, Faculty of Biological Sciences, Kazimierz Wielki University, Chodkiewicza 30, 85-064 Bydgoszcz, Poland; 2 Institute of Dendrology, Polish Academy of Sciences, Parkowa 5, 62-035 Kórnik, Poland

**Keywords:** Local adaptation, Bud-burst phenology, Genotype-environment association, Candidate genes, Sequence capture, Forest tree

## Abstract

**Background:**

Local adaptation is a key evolutionary process that enhances the growth of plants in their native habitat compared to non-native habitats, resulting in patterns of adaptive genetic variation across the entire geographic range of the species. The study of population adaptation to local environments and predicting their response to future climate change is important because of climate change.

**Results:**

Here, we explored the genetic diversity of candidate genes associated with bud burst in pedunculate oak individuals sampled from 6 populations in Poland. Single nucleotide polymorphism (SNP) diversity was assessed in 720 candidate genes using the sequence capture technique, yielding 18,799 SNPs. Using landscape genomic approaches, we identified 8 *F*_*ST*_ outliers and 781 unique SNPs in 389 genes associated with geography, climate, and phenotypic variables (individual/family spring and autumn phenology, family diameter at breast height (DBH), height, and survival) that are potentially involved in local adaptation. Then, using a nonlinear multivariate model, Gradient Forests, we identified vulnerable areas of the pedunculate oak distribution in Poland that are at risk from climate change.

**Conclusions:**

The model revealed that pedunculate oak populations in the eastern part of the analyzed geographical region are the most sensitive to climate change. Our results might offer an initial evaluation of a potential management strategy for preserving the genetic diversity of pedunculate oak.

**Supplementary Information:**

The online version contains supplementary material available at 10.1186/s12864-023-09897-y.

## Background

While the climate changes, species have several possibilities to avoid local extinction due to altered habitat conditions. They can adjust their phenotype (be phenotypically plastic), migrate to a better-suited habitat, or adapt locally to new environmental conditions [1]. Local adaptation results from natural selection and leads to higher relative fitness of resident genotypes in their local habitat than those from other habitats [2]. In the face of climate change, it is essential to test how populations have adapted to their local habitat conditions and predict their future response to environmental change. In particular, for long-lived and sessile organisms such as forest trees, many of which are of ecological and economic relevance, the climate change rate may be too quick for populations to adapt [1].

Local adaptation in forest trees is typically tested through common garden experiments. The correlation between common garden phenotypes and the climatic conditions present at the original site population allows for the identification of potential adaptive traits [3, 4]. Through common garden and provenance experiments, the importance of phenology in facilitating species adaptation to environmental conditions is highlighted by several researchers [5, 6]. They identified variations in the susceptibility of trees to cold and drought, as well as differences in their ability to grow under various conditions [7, 8]. The specific genes which cause the adaptive traits are not revealed through the phenotypic traits exploration [3, 4]. Landscape genomics is useful in investigating the genetic basis of local adaptation and predicting responses of populations to future climate change [9, 10]. It typically relies on two main methods: *F*_*ST*_ outlier tests, which detect differences in allele frequencies between populations, and also genotype-environment association (GEA) methods, which identify adaptive genomic regions and genes by examining correlations between allele frequencies and environmental variables [11]. With knowledge of the genes underlying local adaptation, spatial patterns of adaptive genetic variation as well as background genetic variation can be mapped to identify geographic regions with similar genetic backgrounds [12]. As a result, it is possible to evaluate the scale and pattern of local adaptation in natural populations distributed across a landscape. These approaches complement the fitness and phenotypic variation studies in common garden experiments [9].

Oaks (*Quercus* spp.) are ideal species for studying how forest trees adapt locally to climate change [13, 14]. In the Northern Hemisphere, they are one of the most widespread woody genera, playing an important role in ecological systems [13, 15]. Oaks are expected to benefit greatly from the ongoing climate warming due to their drought resistance and preference for higher temperatures. Therefore, oaks are being considered as a potential replacement for tree species sensitive to drought, such as *Fagus sylvatica* or *Picea abies*, in dry and warm European areas [16]. However, Gentilesca et al. [17] recently described the "oak decline phenomenon" in several European countries due to climate change. It is crucial to comprehend the genetic variation that underlies adaptive traits in populations, and how much of that variation will allow the survival of the species in expected future climate conditions [9].

Previous studies in oaks have used landscape genomic approaches to identify putative candidate genes underlying local adaptation [18–24]. The morphological and genetic differentiation of oak populations have been extensively studied in Europe, and it shows that these differences are influenced by both geographic distance and adaptation [5, 23–27]. Moreover, there is evidence for spatially divergent selection acting on candidate genes related to climate in populations of *Quercus lobata*, suggesting local adaptation [28]. Additionally, an example of adaptive genetic variation related to soil characteristics, local topography, and historical climate was presented in the study of oak species (*Q. petraea., Q*. *pubescens*, and *Q*. *robur*) in Switzerland [23]. In recent times, a few studies have used modeling and mapping of the turnover in allele frequencies of candidate SNPs across present and future climates to explore the relationship between climate and spatial variables and genetic diversity [29–31].

Not much information is available regarding the genetic basis of the timing of bud burst in oaks, as a key target trait in climate change [1, 32, 33]. The phenological cycle of forest tree species can be significantly impacted by rising temperatures. If the temperatures during winter are higher than usual, it could prevent trees from entering dormancy properly if their chilling requirements are not fulfilled during winter [34]. Additionally, if trees start to bud too early because of warmer temperatures and are then exposed to late frosts, it can damage the flower buds and leaves of the trees [35]. Variation in phenological response to climate change is ecologically important because phenology is related to several important factors such as plant physiology, interactions with other organisms in the ecosystem, and the ability to reproduce successfully [36–39].

Differences in the timing of bud bursts were detected among oak species [18, 40]. These differences were found along altitudinal gradients [18, 41] or across the geographic distribution of species [40] in common garden experiments. Similar differences in variation were noticed in other *Fagaceae* species at smaller regional levels [42, 43]. The timing of bud burst has been studied for different oak species by several researchers. For example, Alberto et al. [5] examined the SNPs variation in candidate genes related to bud burst in provenance trials with populations from different altitudinal and latitudinal locations of *Q. petraea*. Using *F*_*ST*_ outlier tests, gene-environment association models and tests of clinal variation of genetic diversity, the researchers detected indications of natural selection for certain genes, which were partially shared across both environmental gradients. In the end, three transcriptomic research studies were carried out to discover the genes responsible for bud burst in oak trees [44–46]. Derory et al. [47] discovered 19 QTL (Quantitative Trait Loci) which have impact on the timing of bud burst in *Q. robur.* Studies were started to investigate candidate genes to find indicators of natural selection and to find genetic markers that factor into bud burst. Another method could take a closer look at sets of genes that are believed to play a role in adapting to changing climate conditions [3, 5, 48, 49]. This approach has an advantage in that the functional genes and their variants can be directly associated with regulating a portion of the observed adaptive phenotypic variation [3, 50]. Based on this rationale, we recently demonstrated that in beech (*Fagus sylvatica*) the genetic diversity of genes related to bud-burst phenology was affected by environmental conditions, thereby facilitating local adaptation [51].

Here, we used two complementary methods: a candidate gene approach and direct phenotypic evidence from a common garden experiment to investigate the local adaptation to current and future climate in pedunculate oak (*Quercus robur* L.) in Poland. We focused on candidate genes that were found to be differentially expressed during bud burst in European oaks (*Q. robur, Q. petraea*) [45]. We used a sequence capture approach to create a dataset of SNPs to: (i) detect spatial patterns of genetic diversity based on geographic variables; (ii) discover SNPs related to local climate; (iii) find associations between adaptive characteristics and genetic polymorphisms, such as individual and family spring and autumn phenology scores, survival rate, and tree diameter at breast height (DBH) and height, measured at the location of a common garden experiment; (iv) map putatively adaptive variation to evaluate the extent and pattern of local adaptation. We employ genotype-environment association and differentiation-based outlier tests to identify putatively adaptive SNPs and then use a machine-learning approach to collectively evaluate support for putatively adaptive SNPs and map adaptive allele frequency changes on the landscape.

## Results

### Phenotypic variables

The results of the canonical correlation analysis demonstrated significant relationships between measurements of physical traits and either geographic or climatic variables both at the individual and family levels (Table S1). Within individual phenotype variables, it was found that only individual spring phenology was moderately correlated with latitude (*r* = 0.289; *p* = 0.007) and also BIO1 (*r* = 0.248; *p* = 0.020), BIO2 (*r* = -0.247; *p* = 0.021), and BIO8 (*r* = 0.357; *p* = 0.001). In contrast, family phenotypic measures were strongly correlated with most geographic and climatic variables (Table S1).

## Sequence capture data

An average of 4,724,562 raw sequence reads for each individual were produced and over 98% of reads were successfully aligned to the pedunculate oak genome assembly PM1N [52]. The sequencing data which corresponds to distinct positions in the genome were analyzed to detect SNPs among individuals, in the result 366,252 unfiltered SNPs were identified. After the application of the filtering process described in the Materials and Methods section, the initial set of 189,005 SNPs (before LD pruning) was reduced to 97,527 SNPs by performing linkage disequilibrium (LD) pruning.

We successfully identified 720 well-defined gene models from the initial set of 1732 candidate genes. These genes comprised 428 complete and 292 partially valid gene models (without untranslated regions). The cumulative sequence length was equal to 2,568,166 bp (Table S2). Then, we used 18,799 SNPs (mean coverage 65x) located either within or up to 500 bp from the set of 720 gene models. We found that 12,680 (67.45%) out of the total 18,799 SNPs were located within the candidate genes themselves, with an average density of 4.9 SNPs/Kbp (or one SNP per 203 bp). The remaining 6,119 SNPs (32.55%) were identified within 500 bp from gene models (average distance of 214 bp). Based on the gene models annotation: 7413 (39.43%) were found in exons; 6678 (35.52%) in coding regions (CDS); 290 (1.54%) in five prime untranslated regions (5’UTR), 445 (2.37%) in three prime untranslated regions (3’UTR), and finally 5267 (28.02%) in introns (Table 1). The dataset had a very low level of missing data, with an average of 180.63 SNPs (0.96%) per individual, corresponding to only 27 to 885 missing loci per tree.

**Table 1:** Number and percent of 18,799 SNPs located in different genomic regions

Category	Number of SNPs	%
Exon	7413	39.43
CDS	6678	35.52
5’ UTR	290	1.54
3’ UTR	445	2.37
Intron	5267	28.02
Unclassified	6119	32.55

### Genetic structure and outliers

**Fig. 1:**
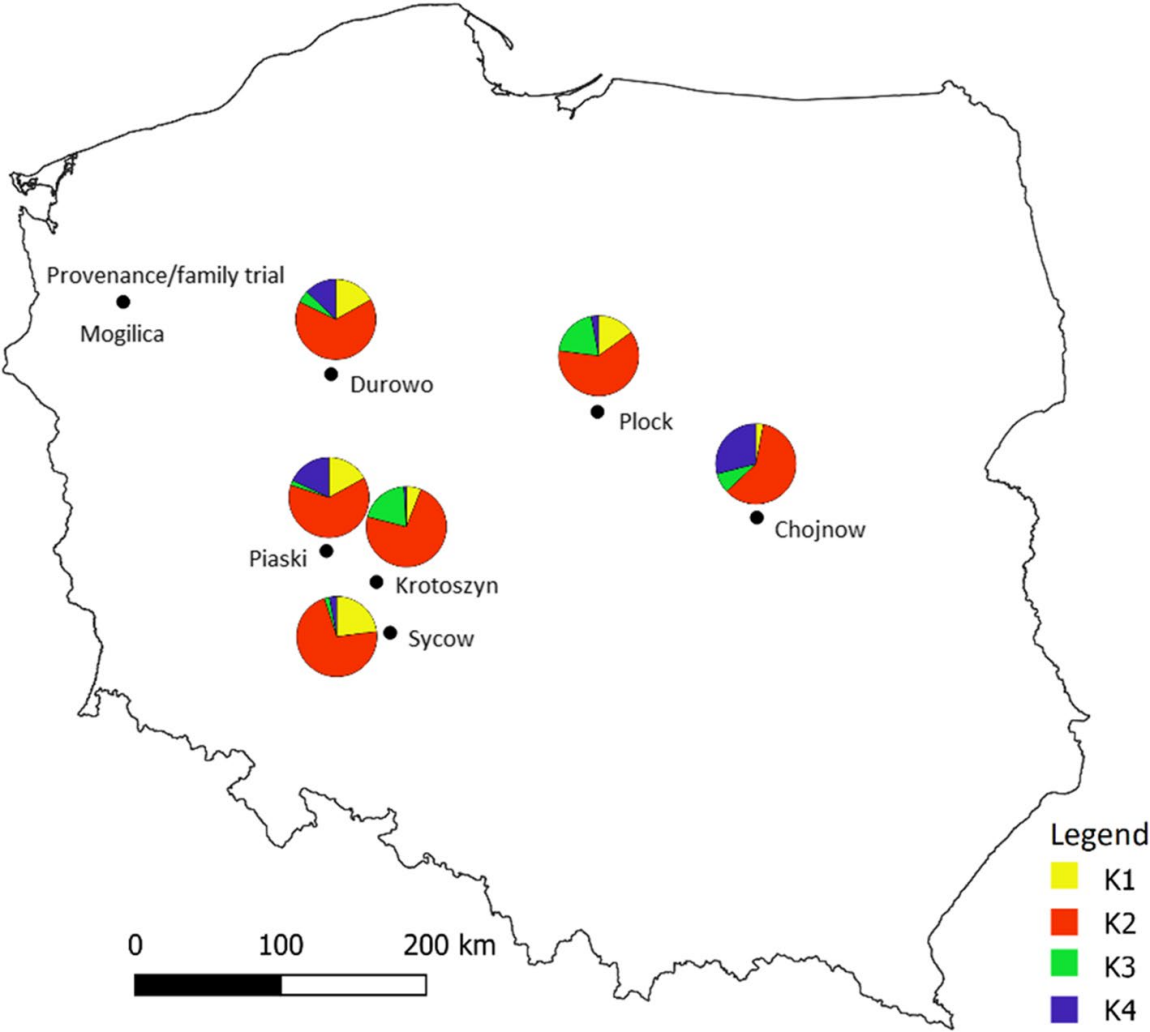
Genetic structure of *Q. robur* in Poland. The pie charts show the proportions of membership of each population for the estimated value of K = 4 genetic groups

**Fig. 2:**
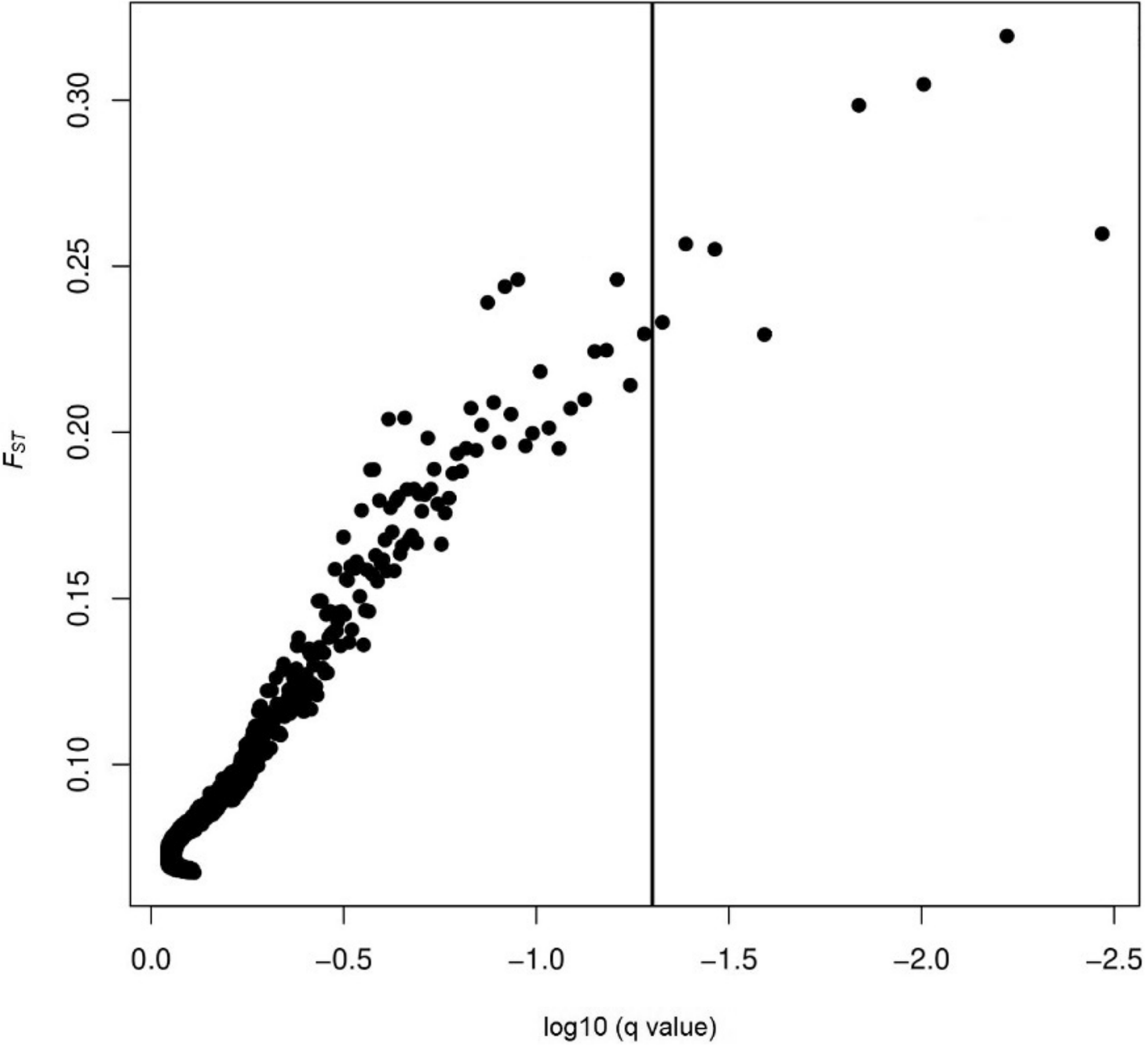
Results for the differentiation ( *F*_*ST*_) outlier test based on four gene pools of *Q. robur* in Poland. SNPs with log_10_*q* less than -1.3 (corresponding to *q*-value < 0.05) are considered outliers

The average nucleotide diversity of phenology-related genes in the dataset of SNPs before LD pruning (189,005 SNPs) for all populations ranged from 7.97 × 10^–5^ to 0.046, with a mean equal to 2.88 × 10^–3^. The Bayesian clustering implementation done in STRUCTURE reveals the existence of four distinct gene pools (Fig. S1). A graphical representation of membership to clusters for *K* = 4 is shown in Fig. S2. The distribution of gene clusters in the landscape did not show any geographical structure (*p* > 0.130) (Fig. 1). BayeScan identified 8 SNPs (0.01% of 18,799 SNPs), all located within genes (as shown in Table S3) with elevated *F*_*ST*_, which indicative of divergent selection (Fig. 2). The average *F*_*ST*_ value for the identified outliers was 0.270, with the range from 0.229 to 0.319.

### Environmental association analysis

The LFMM analysis identified 781 unique SNP loci (4.34% of the total SNPs) that exhibited a significant association with at least one variable, as shown in Table S3. The highest number of associations (980) involving 577 unique SNPs were found for family phenotypic variables (adaptive traits): spring phenology (169), autumn phenology (281), DBH (188), height (147), and survival (195). Next, 477 associations (with 316 SNPs) appeared to be related to climate variables: BIO1 (69), BIO2 (48), BIO8 (113), BIO11 (58), BIO12 (107), and BIO14 (82). Next, 288 associations (276 SNPs) were related to individual phenotypic variables: spring (53) and autumn (235) phenology. Finally, 126 associations (120 SNP loci) were linked with geographic variables: longitude (38) and latitude (88). The highest number of shared SNPs were discovered for family DBH and height (135), individual and family autumn phenology (117), family DBH and autumn phenology (79), and BIO12 and family autumn phenology (74). Combined, BayeScan and LFMM identified 789 candidate SNPs (Table S3). Interestingly, there were no common SNPs between the two datasets of SNPs; however, four genes, including Qrob_T0700410.2 (conting OCV4_rep_c39832, OCV3_prime_rep_c56859), asmbl_334 (conting OCV4_rep_c22203), asmbl_534 (conting OCV4_rep_c28592), asmbl_676 (conting Loc_104743_Tr_1/1_Conf_1.000_Len_2681), had SNPs identified by both methods. The genetic differentiation between populations was estimated for each adaptive dataset of SNPs. The highest *F*_*ST*_ value was found in the *F*_*ST*_ outliers dataset (0.001) and also sets of loci associated with geography (9.97 × 10^–3^) and phenotypic traits (9.63 × 10^–3^) (Table 2).

**Table 2:** Summary of gradient forest analysis

SNP datasets	Number of SNPs	*F*_*ST*_	SNPs with *R*^2^ > 0 (%)	Mean *R*^2^ (%)
All	18799	6.51 × 10^–3^	938 (4.99%)	4.08
Geography-associated loci	119	9.97 × 10^–3^	5 (4.20%)	2.88
Climate-associated loci	316	9.41 × 10^–3^	15 (4.75%)	3.69
Phenotypic traits-associated loci	672	9.63 × 10^–3^	34 (5.06%)	4.63
*F*_*ST*_ outliers	8	0.001	0	-

### Genomic contexts of candidate SNPs

The gene models of *Q.* *robur* (Table S2) were used to determine the genomic contexts of the 789 identified candidate SNPs. Our analysis revealed that 516 SNPs were located within 287 genes, with 283 (54.8%) of these SNPs as CDS variants. Here, 210 SNPs (74.2%) were non-synonymous. Another 273 SNPs were found in intergenic regions (also shown in Table S3). Of the 287 genes, 162 had annotations in the *Q. robur* genome. These genes encoded proteins involved in various biological processes: response to stresses (i.e., cold, light stimulus, salt stress, water deprivation, and bacterium), cellular metabolic processes (lignin catabolic processes and protein-chromophore linkage), cell division, protein folding, organic substance metabolic processes (lipid and pectin catabolic process sand carbohydrate metabolic processes) gene expression (translation and transcription), cell growth, developmental processes (cuticle and flower development) and photosynthesis (see Table S4).

### Current and future predictions of adaptive genetic variation

Initially, the GF model was applied to the entire dataset of 18,799 SNPs, followed by the three adaptive SNP datasets identified through LFMM analysis, and finally the eight *F*_*ST*_ outliers (Table 2). Among the adaptive GF models, the model for the phenotypic traits-associated loci explained variation the best (mean *R*^*2*^ = 4.63%) when compared with the model for a complete dataset of 18,799 SNPs (mean *R*^*2*^ = 4.08%). *F*_*ST*_ outliers GF model found no significant relationship between allele frequencies and spatial or environmental variables.

**Fig. 3:**
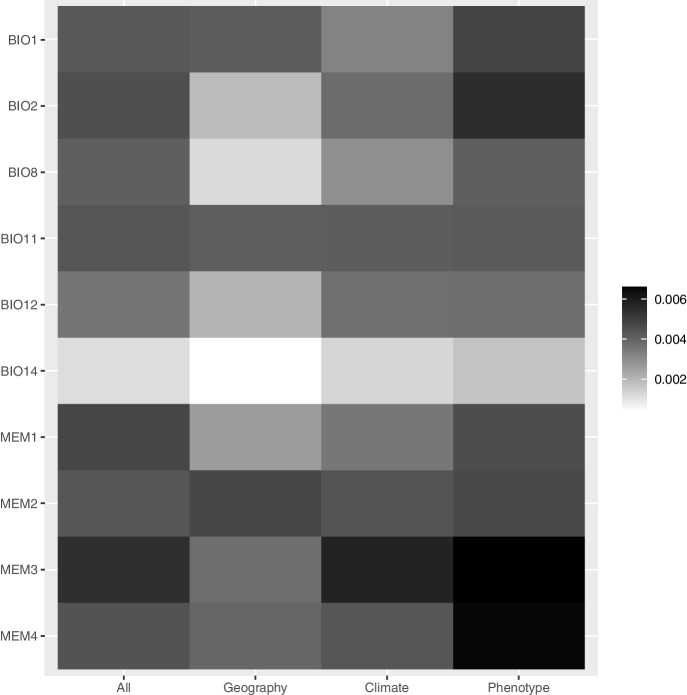
The relative importance (*R*^*2*^) of the predictor variables used in gradient forests (GF) for the four SNP sets

In terms of variable importance, spatial variables (MEMs) were the most important predictors for all significant GF models (Fig. 3), indicating a strong influence of geographical location on the change of allele frequency across the landscape. After summing the variables' importance, the four MEM variables explained about 48.54% of the model variation, and climate variables explained the remaining 51.46% in all GF models. Besides MEM variables, the temperature variables were the most important environmental predictors. Mean diurnal range (BIO2) was the third most important predictor after two spatial variables (MEMs) for all SNP and phenotypic traits-associated loci GF models. In addition, the geography GF model found annual temperature (BIO1) to be the most important environmental variable after the second eigenfunction (MEM-2). On the other hand, the climate SNP GF model revealed that the mean temperature of the coldest quarter (BIO11) was also an important environmental variable explaining the changeover of allele frequencies (Fig. 3).

Patterns of turnover in genetic composition varied by SNP dataset and by spatial or environmental gradients (Fig. S3). For each spatial environmental predictor, the aggregate turnover functions from GF were similar in shape and magnitude for all SNP datasets (Fig. S3) with the most notable exceptions to this pattern being the prominent response of adaptive GF models to annual mean temperature (BIO1) and mean temperature of coldest quarter (BIO11). The adaptive GF models showed a strong threshold response near values of mean annual temperature between 8.08 and 8.15 °C, and also of the mean temperature of the coldest quarter between -1.4 and 1.2 °C.

**Fig. 4:**
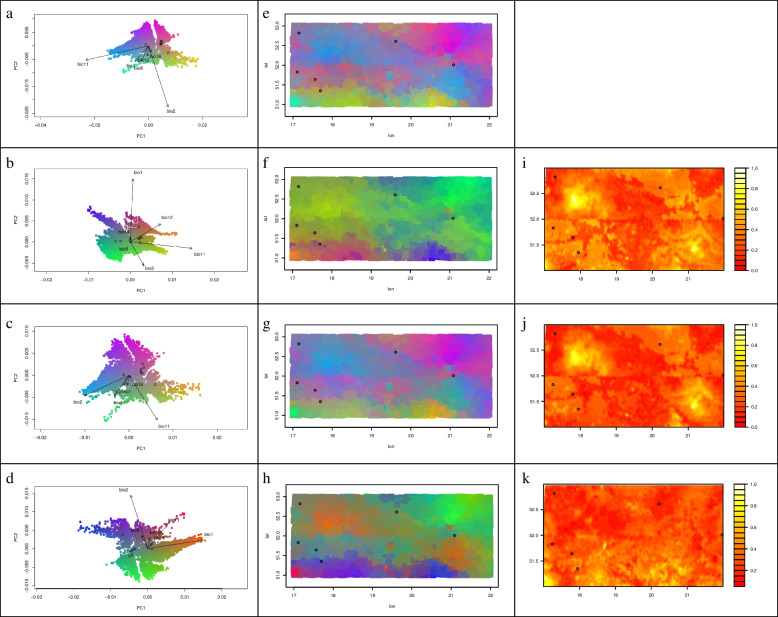
Projected spatial turnover of *Q. robur* allele frequencies using GFs for different subsets of SNPs: all SNPs (**e**); SNPs associated with geography (**f**); climate (**g**); and phenotypic traits (**h**). The biplots (**a**-**d**) indicate the contribution of the environmental variables to the predicted patterns of genetic turnover (**e**–**h**), with labeled vectors indicating the direction and magnitude of environmental gradients. The difference between GF models (**i**-**k**) mapped in (**e**) and (**f**–**h**) is based on Procrustes residuals, scaled by the maximum distance found for each comparison. Black circles (**e**-**k**) indicate the locations of genotyped populations

When mapped in geographic space, the patterns of the genetic composition of all SNPs predicted by GF (Fig. 4e) and the subsets of SNPs related to climate (Fig. 4g) and phenotypic traits (Fig. 4h) were similar, with rapid turnover predicted in a belt from northwest to southeast part of analyzed geographical region. All SNPs and a subset of SNPs related to climate GF models identified temperature variables (BIO2, BIO11) as major environmental correlates of these patterns (Fig. 4a, c). However, a geography related dataset revealed the importance of temperature (BIO1, BIO2, BIO11) and, to lesser extent, annual precipitation (BIO12) variables (Fig. 4b). Nevertheless, BIO1 and BIO2 explained a large proportion of variance in the phenotypic traits dataset (Fig. 4d). The mapped pattern for SNPs associated with geography (Fig. 4f) as compared to reference SNPs was less congruent, although predicted patterns from SNPs associated with geography model exhibited greatest turnover in the eastern part of the analyzed geographical region and comparatively less in the southeast region. Here BIO1 and BIO11 explain more variance in the subset of SNPs related to geography than in the all SNPs dataset (Fig. 4d). The difference in predicted patterns of allele turnover between all SNPs and the subsets of SNPs related to climate (Fig. 4j) and geography (Fig. 4i) were greatest and most extensive in the northwest and southeast part of analyzed geographical region, as indicated by the mapped Procrustes residuals. For the phenotypic traits SNP dataset (Fig. 4k), the predicted pattern of turnover in genetic composition differed from all SNPs in the south part of the analyzed region. The areas that appear more yellow in Fig. 4i-k indicate a greater difference in adaptive genetic variation in comparison to the overall genetic composition.

**Fig. 5:**
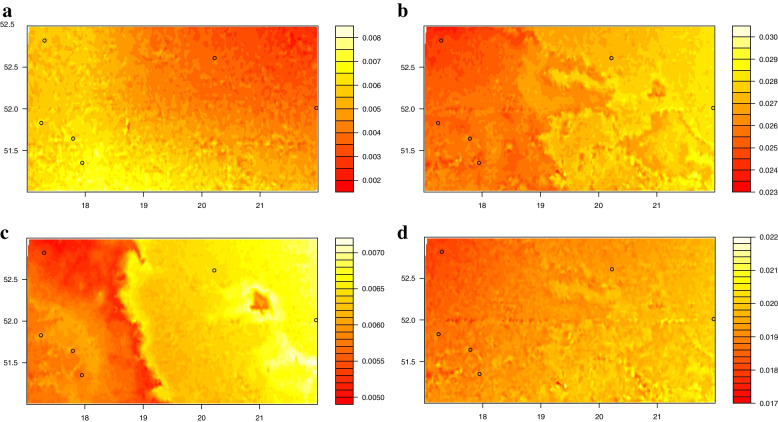
Predicted genetic offset for the complete dataset of SNPs (**a**) and the subset of SNPs related to geography (**b**), climate (**c**), and phenotypic traits (**d**) under the climate change scenario for 2080. Black circles (**a**-**d**) indicate the locations of genotyped populations

The GF future predictions for subsets of SNPs associated with geography (Fig. 5b), climate (Fig. 5c), and phenotypic traits (Fig. 5d) showed that the largest genetic offsets under climate change are expected in the eastern part of the analyzed geographical region. Similarly, the western part of the range was predicted by these models to experience a relatively low genetic offset between current and future climates. For all SNPs, GF identified that most of the western region has a relatively high genetic offset. Predictions from GF showed nearly the opposite pattern for all SNPs, with predicted genetic offset much smaller and less widespread than for three adaptive SNP datasets (Fig. 5a).

## Discussion

In the present study, we investigated the adaptive genetic variation of pedunculate oak (*Q. robur*) in Poland and attempted to predict their fate under the scenario of climate change. By combining the candidate genes approach with geography, climate, and phenotypic data (Table S1) in six populations (Fig. 1), we gained insight into the patterns of adaptive variation within this species. We detected SNPs at phenology-related genes that are putatively involved in local adaptation and we identified the factors that are potentially driving this process. We then combined the allele frequency data of these candidate SNPs with the present and future climatic data to estimate the potential adaptation of the studied populations under future climate change.

### Genetic diversity and population structure

The present study discovered 18,799 SNPs in 720 phenology-related genes. Nucleotide diversity (*π*) in the dataset before LD pruning (189,005 SNPs) ranged from 7.97 × 10^–5^ to 0.046 with a mean equal to 2.88 × 10^–3^. Only several studies have examined nucleotide diversity in genes of oak species until now. One of them is done by Derory et al. [47] who examined the diversity of eight bud burst-related genes in *Q. petraea* and Quang et al. [53] who studied 11 genes in *Q. crispula* in Asia. Comparable levels of diversity were found in both studies, *π* is respectively 6.15 × 10–3 and 6.93 × 10–3. However, Homolka et al. [20] found a slightly lower diversity of genes related to drought response in *Q. petraea* (*π* = 3.74 × 10^–3^) as in *Q. robur* (π = 3.65 × 10^–3^). The observed nucleotide variation can be significantly affected by the geographical distribution of the sampled populations. Additionally, the nucleotide diversity may differ significantly between different populations.

The genetic structure revealed weak differentiation (*F*_*ST*_ = 6.51 × 10^–3^) between the studied populations (Table 2). Individuals can be assigned to four genetic clusters, but almost 70% of them belong to cluster 2 (Fig. S2). In addition, the distribution of gene clusters in the landscape did not show any geographical structure. The phylogenetic structure of white oaks in Poland has been discussed in previous publications, which revealed three clades related to the major maternal lineages (Iberia, Apennines, Balkans) [54, 55]. However, most individuals belonged to the Balkan maternal lineage (71.5% in Dering et al. [54], and nearly 64% in Chmielewski et al. [55]), which is likely reflected in this study. Contrary to the extensive research on the chloroplast genome, there is a limited number of studies on the nuclear genome's genetic differentiation of pedunculate oak. The study done by Degen et al. [56] discovers weak but the clear genetic structure of pedunculate oak populations in Europe using nuclear markers. They identified two gene pools, with the first gene pool being more prevalent in the west, and the second gene pool being more dominant in the east. The phylogenetic pattern of genetic structure (south-to-north direction) was modified when nuclear gene markers were used. This change was due to pollen-mediated gene flow and introgression from *Q. petraea* in a west-to-east direction.

### Signals of selection and association analysis

The results of this study provide strong evidence of selection. The BayeScan analysis identified eight outlier loci with a mean *F*_*ST*_ value equal to 0.270. Studies have shown that BayeScan is typically effective in identifying a large proportion of true selective loci and has a low rate of false-positive outliers (less than 1%) [57, 58]. In addition, the presence of complex demographic patterns could lead to false-positive evidence of selection [57, 58]. However, our sampling design included geographically close populations, that have a similar genetic structure, but thrive in different environmental conditions and are differentiated by selection. Therefore, the low number of outliers in this study provides a reliable set of candidate loci under divergent selection. Similar results were reported in oaks. For example, Homolka et al. [20] found only six outliers in drought-related genes in European oaks in Austria, while Vanhove et al. [31] found 11 outliers for *Q. suber* in its whole natural range in Europe. The intense gene flow in oaks may account for the comparatively small number of outlier SNPs, which could potentially counteract responses to relatively weak selective pressures [25].

Environmental association analyses, which are considered more powerful than genetic differentiation tests [57], identified 781 unique SNPs in 385 phenology-related genes in our dataset. These SNPs were found to be significantly correlated with at least one variable related to adaptive phenotypic traits, climate, or geography. This method has the benefit of being able to determine the specific environmental drivers of local adaptation. Experiments in plants, including forest tree species (e.g., *Betula*, *Picea*, *Populus, Quercus*, *Salix*, and *Fagus*) have shown that the timing of bud burst is affected by temperature regimes [59–62]. In this study, most of the SNPs (216 unique SNPs) that were related to climatic variables were found to be correlated with temperature variables.

The highest number of SNPs related to temperature than to precipitation has also been identified by other studies [19, 63–66]. The association of SNPs with temperature and precipitation variables in oaks differs depending on the species. For instance, Rellstab et al. [19, 23] reported that most of the significant SNPs were associated with precipitation variables in *Q. pubescens* and *Q. robur* populations in Switzerland. In contrast, temperature variables had most of the associations in *Q. lobata* [19, 23] and *Q. petraea* [19, 23]. Both precipitation and temperature may play a significant role in the selection, but their effects may vary depending on which climate variables are more crucial in different environments and geographic regions, ultimately affecting different sets of genes within populations [28, 51].

An alternative method to identify genomic signatures of selection is by conducting association tests between genotypes and traits in populations that are grown in a common garden experiment [67]. Such genotype–phenotype association analyses have many advantages, in that they allow for the explanation of the links between genes (or genomic regions) and traits that could potentially be under selection [68]. A total of 672 unique SNPs showed significant association with phenotypic variables of pedunculate oak (individual and family spring and autumn phenology, family DBH, family height, and family survival). The highest number of SNPs were related to family (577 unique SNPs) and individual (276 unique SNPs) autumn phenology. Among them 117 SNPs were common. The higher number of loci related to family than individual phenology is probably related to using the means of multiple measurements of adaptive traits of mother trees, which resulted in higher measurement accuracy.

The knowledge of the environmental control of autumn phenology in forest tree species is limited. Earlier research on the environmental factors influencing autumn phenology has concentrated on the impacts of frost, autumn chilling, day length, and precipitation [69–73]. Here, we observed a positive correlation between family autumn phenology and annual temperature (BIO1) and a negative correlation with annual precipitation (BIO12). We can conclude that warm temperatures and less precipitation lead to earlier autumn phenology in pedunculate oaks. This is in line with a previous study that showed both oak and beech species can avoid severe drought stress by initiating leaf senescence earlier, which prevents the loss of resources and nutrients caused by drought-induced leaf death [74].

Lower numbers of loci have been associated with the individual (53 SNPs) than family (169 SNPs) spring phenology. However, these numbers are still higher than those reported by Derory et al. [47], who found 19 QTLs associated with bud burst in *Q. robur* in France. In addition, Alberto et al. [5, 75] found only 15 genes related to variation in bud burst in *Q. petraea* through provenance trials with populations sampled from altitudinal and latitudinal gradients. This may suggest that despite the populations being sampled over such a small geographic area, the inter-population differences in the timings of bud burst might be significant for *Q. robur*.

There was also a low (individual) to non-existent (family) correlation between spring and autumn phenology, reinforcing previous studies showing that the genes [76] and environmental factors [77] underlying spring and autumn phenology are different. Both individual and family spring phenology had positive correlations with survival at the family level, suggesting that earlier bud burst is beneficial for tree survival. Also, there was a stronger correlation between bud burst and growth (DBH, height), suggesting that earlier bud burst was beneficial for growth.

Association analysis between genotype and geographic variables revealed a stronger relation of phenology-related genes with latitude (88 SNPs) than longitude (38 SNPs). In addition, individual spring phenology was positively correlated with latitude (*r* = 0.289; Table S1). This pattern contrasts with previous studies of oaks [78, 79], which showed that the timing of bud burst varies with latitude, with populations in the south exhibiting earlier leafing than those in the north. However, our results are consistent with Jensen's findings [80] in comparative trials of sessile and pedunculate oaks, where it was observed that northern provenances flushed earlier than southern provenances.

It is surmised by Farmer and Reinholt [80] that earlier bud break in trees might be linked to earlier but set (i.e., increasing the length of winter dormancy), as this would act to change the depth of bud dormancy by providing more time for accumulating chilling requirements. This explanation is possible for pedunculate oak in Poland, but bud-break results from our trials included genotypes originating from a relatively restricted range of latitudes (51–52°N).

### Genes underlying variation in bud burst

This research detected 789 candidate loci that could be under selection, with 410 of them located within functional genes that are annotated in the *Q. robur* genome. These genes play a role in various physiological processes such as the transport of ions and proteins, metabolic and developmental processes; regulation of transcription and translation; and response to abiotic and biotic stress. Here, we focused solely on genes related to spring and autumn phenology.

Desiccation stress is experienced by plants during the dormancy stage, which can be seen by the discovery of late embryogenesis abundant protein gene (LEA) being related to individual and family autumn phenology. Indeed, although its biological function remains unclear [81], genes encoding LEA proteins have been isolated in various plant tissues under moisture stress. On the other hand, we found heat-shock genes (e.g., heat shock cognate protein 8, heat shock protein 83-like, and stromal 70 kDa heat shock-related protein) associated with spring phenology that accumulates at the onset of flushing, the accumulation of heat-shock proteins probably protects cells from temperature stresses [45].

The galactinol synthase gene association with autumn phenology is also related to the onset of bud burst [82, 83]. Galactinol synthase is responsible for an initial step in the production of raffinose family oligosaccharides (RFOs) from UDP-galactose. These RFOs are involved in the desiccation tolerance of seeds, and galactinol synthase has already been previously reported to have a role in enhancing the plant's ability to tolerate heat and drought stresses [82–84].

Genes of histones, as well as transcription factors, are induced at the onset of bud burst. We found histone H3 and histone H3-like centromeric protein CSE4 were associated with spring phenology, which seems to reflect increased cell division activity. Some indicators of developmental processes taking place during bud burst might be provided by the induction of some transcription factor genes. For example, GATA transcription factor 9-like is related to flower and shoot apical meristem development [84]. Similarly, ethylene-responsive transcription factor WIN1-like, associated with autumn phenology, probably increases the rate of cell division in the apex. This transcription factor was described as an important regulator of the bud-break process in transgenic poplar plants [85].

Genes involved in energy metabolism in cells may be specifically expressed during the final stages of bud burst, as shown by genes of cytochrome P450 71A1-like or CAB6 (chlorophyll a/b-binding protein 6). In addition, the glyceraldehyde-3-phosphate dehydrogenase gene was associated with the family autumn phenology. This is probably related to the activity of enzymes involved in the glycolytic pathway increasing at the release of dormancy [86].

Nevertheless, several unknown genes described as “hypothetical protein” or “uncharacterized protein” were also associated with spring and autumn phenology. The putative role of these genes in the control of bud burst remains to be determined.

### Current and future predictions of adaptive genetic variation

The strongest driver affecting the change in allele frequencies in pedunculate oak in Poland were geographic variables (MEMs) (Fig. 3). The reason why the spatial variables take so an important role might be due to the spatial genetic structure, however, it could also indicate the presence of important unmeasured environmental predictors that MEM might capture [29, 87], including the effects of human-mediated translocations of forest reproductive material in the past [88]. After MEM variables, temperature predictors were the most important environmental predictors for all GF models (Fig. 3). Consequently, for all SNPs (Fig. 4e) and the subsets of SNPs related to climate (Fig. 4g) and phenotypic traits (Fig. 4h), the predicted change of allele frequencies across the landscape was similar with rapid turnover predicted in a zone from northwest to southeast part of analyzed geographical region (Fig. 4 e, g, h). The mapped pattern for SNPs associated with geography (Fig. 4f) as compared to reference SNPs was less congruent, although predicted patterns from SNPs associated with the geography model exhibited the greatest turnover in the eastern part of the analyzed region. It is not surprising that an important role in GF models plays spatial variables given that most plants show spatial autocorrelation due to isolation by distance. For instance, a graduate gradient has been found in GF models for *P. balsamifera* by Fitzpatrick and Keller [12]. Furthermore, the strong spatial structure of *Acacia koa* populations in Hawaii Island has been found by Gugger et al. [89].

The similarity among GF models observed in the maps of Procrutes residual is explained by the strong spatial influence (MEMs). They were mostly a bit higher in the northwest and southeast part of the analyzed geographical region meaning that populations in these regions are potentially adapting to a changing environment (Fig. 4 i, j). Nonetheless, minor differences between adaptive SNP datasets and GF models for all SNPs also illustrate that the spatial pattern of genetic diversity is being shaped by the climate environment.

In three adaptive models (Fig. 5b-d), the predictions for future relationships between genes and the environment suggest that the populations in the eastern part of the analyzed geographical region (Płock and Chojnów) are likely to experience a significant disruption (more yellow colors in Fig. 5). Without adaptive evolution or migration, trees in these areas are projected to become less adapted to future climate conditions over time. Western populations are adapted to the higher annual temperatures and precipitation seasonality (Table S1), and would likely accommodate a greater chance of those parameters than eastern ones. It should be emphasized that the evolutionary response of these populations to climate change will be more complicated than these estimates because adaptation is shaped by multiple evolutionary processes such as mutation, migration, recombination, and the effective population size [12].

The match between new environments and genotypes is going to play an important role in the forest tree population’s future. Scientists around the world are trying to address these questions through common garden experiments and also new phenotyping and genomic approaches as well as using traditional common garden approaches to estimate the ability to adapt to new climates and inform assisted gene flow (AGF), the managed translocation of individuals within the current species range to facilitate rapid adaptation to climate change [89]. Our findings demonstrate that landscape genomics can detect areas that could profit from AGF [89]. For example, in the eastern part of an analyzed region, it may be suitable to introduce seeds from the western part of the same region because these seed sources are likely to contain genotypes that are already preadapted to the predicted future climatic conditions. Unfortunately, we know very little about the efficiency of AGF in pedunculate oak. On the other hand, the high potential for long-distance gene flow of pedunculate oak via pollen movement [90] could increase genetic connectivity [91] and contribute to maintaining sufficient genetic variation in the face of climate change.

## Conclusions

The present study demonstrates a weak genetic structure of pedunculate oak populations in Poland. Environmental association analyses revealed that temperature variables were more frequently associated than precipitation variables with polymorphisms at phenology-related genes. Through gradient forest analysis, it was possible to identify the factors responsible for the genomic variation within the species. Moreover, the study showed that eastern regions of the pedunculate oak distribution in Poland are susceptible and at risk of climate change. The additional sampling is necessary to validate the identified gene associations and to create a broad view of the distribution of adaptive genetic variation for the basis of experiments that could evaluate the sensitivity of seedlings transferred into present environmental conditions with the expectation that they will be adapted to future climates. Such tests could check whether the current genetic variation is sufficient to tolerate future climate conditions or whether any practices, such as assisted gene flow, would improve the resilience of forest ecosystems.

## Materials and methods

### Plant material

The individual samples used in the study were collected from the provenance/family common garden trial of *Q. robur* established in Forest District Mogilica in north-western Poland (Fig. 1, Table S5) by the Institute of Dendrology of Polish Academy of Sciences (Kórnik). The seeding material used to establish the trial originated from 186 mother trees in Poland, which are growing in eight forest seed stands (considered here as provenances). From each mother tree, seeds were collected from the ground under the canopy of the tree. Each mother tree (family) was represented by an average of 45 offspring giving a total of 8,640 individuals in the trial.

In this study, we sampled 5 to 31 individuals (87 in total) of pedunculate oak originating from 6 provenances (geographic and climate data points) selected based on two main criteria: (1) high phenological variability of the offspring within and between families; (2) diversity of climatic conditions in locations of the forest seed stands. The freshly developed leaves were collected from individuals in the early spring of 2015.

### Acquisition of environmental information

Climate data of the six provenance locations were obtained from the WorldClim database [92] (http://www.worldclim.org) from maps with a spatial resolution of 30 arc-seconds (approximately 1 km^2^) using DIVA-GIS 7.5 software [93]. The highly correlated data (ǀ*r*ǀ > 0.70; [94]) was removed, thus, the set of climate variables consists of annual mean temperature (BIO1), mean diurnal range (BIO2), mean temperature of the wettest quarter (BIO8), mean temperature of coldest quarter (BIO11), annual precipitation (BIO12), precipitation seasonality (BIO14) (Table S5).

### Phenotypic variables

An individual and family phenotypic index was assigned to each collected individual (Table S5). The individual spring (five points scale) and autumn (eight points scale) phenology were measured in 2008. However, family scores of spring and autumn phenology were assessed in 2005, while DBH, tree height, and survival were measured in 2006. This group of traits is considered and referred to as ‘phenotypic traits’ in this study. Family indicators represent average measures of the observations for offspring of each mother tree. The relationships between variables were scrutinized based on pairwise Pearson’s correlation and canonical correlation analyses (using STATISTICA software).

### DNA extraction and sequencing

Total DNA was extracted and quantified using methods reported by Meger et al. (2021). We assessed the genetic variation of individual trees using the exome capture approach [95] based on the set of initially selected 1732 candidate genes (Table S4). Firstly, we chose the set of 1732 genes using the previously published *Quercus* transcriptome (OCV3, [45]). The genes had been identified by the EdgeR method by Lesur et al. [46] as having diverse expressions during the release of bud dormancy. Secondly, based on literature surveys, we chose an additional 103 candidate genes which have been selected in other species targeting genes of putative adaptive importance associated with photoperiod (20) and drought stress (83). The selected sequences of *Fagus sylvatica* [96], *Populus trichocarpa* [97, 98], and *Pieca abies* [99] were inspected by BLAST 2.0 software from Washington University to find corresponding homologous sequences in the oak transcriptome (OCV3, [45]). We aligned our sequences against GenBank’s non‐redundant protein database (NR), TrEMBL, and UniProt Swiss‐Prot protein databases, using Blastx with an e-value cutoff of 0.0001. The information on matching sequences was utilized to annotate candidate genes. We obtained gene names, general descriptions, and Gene Ontology (GO) categories, along with other relevant information from the UniProt database for significant matches.

The DNA library preparation and sequence capture were carried out using recommended methods in the SeqCap EZ Library SR User’s Guide v5.1 (Nimblegen Roche). Since the reference genome of *Q. robur* was not available at the first stage of the experiment, the bait development process used in SeqCap EZ library preparation employed only cDNA sequences of the 1732 genes [45]. Following capture, the 125 bp paired-end sequencing of 87 samples was performed on a single lane of an Illumina HiSeq2500 sequencer. Sequence capture and sequencing were performed by IGA Technology Services (www.igatechnology.com; Udine, Italy).

Sequence reads were assessed with FastQC software (Andrews, 2010; available at http://www.bioinformatics.babraham.ac.uk/projects/fastqc/). Adapter sequences were removed with Cutadapt [100], and contaminant (e.g., chloroplast genome) sequences were removed with ERNE-FILTER [101]. Purified high‐quality reads were mapped to the pedunculate oak genome assembly PM1N [52] using Burrows-Wheeler Aligner (BWA-MEM algorithm) with default settings [102, 103]. SAM files were converted to BAM files, sorted, and indexed using SAMtools v.0.1.19 [104]. Taking a minimum Phred-scaled confidence threshold of 30, SNPs were identified by GATK 3.5 [105]. Next, the tools from GATK: “SelectVariants” and “VariantFiltration”; were used to exclude low-quality variants by applying the following filter settings MQ < 40.0, QD < 20.0, ReadPosRankSum < -8.0, and MQRankSum < -12.5. Filtering of variants (with minimum coverage > 10x), LD pruning and SNP classification was performed as described in our previous paper [51].

### Genetic structure and outliers

The nucleotide diversity (π) of candidate genes in the dataset before LD pruning (189,005 SNPs) was calculated using VCFtools [106]. The genetic structure of the sampled individuals was assessed using STRUCTURE 2.3.4 software [105]. The number of tested populations (*K*) was set from 1 to 10, each analyzed in 10 repeats, with 50,000 burn-in iterations and 100,000 MCMC repeats, and an admixture model with correlated frequencies. *ΔK* method [107] implemented in the software STRUCTURE HARVESTER 0.6.94 [108] was used to identify the optimal value of *K*. The genomic regions under spatially divergent selection were identified based on the STRUCTURE clusters using the Bayesian approach in BayeScan 2.1 [109], it is widely recognized as the most efficient method for population differentiation [57, 58]. The analysis was performed with 20 pilot runs with 5,000 iterations and 500,000 iterations MCMC, a burn-in of 50,000 iterations and a prior odds ratio of 10. To assess the probability of the model with selection compared to the neutral model, *q*-values were used [110] which are posterior probabilities that have been adjusted for multiple tests. We defined outliers as SNPs with a q-value less than 0.05 (-log10q > 1.3).

### Environmental association analysis

To identify candidate SNPs that may be under the influence of natural selection, we used latent factor mixed modeling (LFMM; [111]) as implemented in the LEA package [112] in R to test for significant associations of geographic, climate, and phenotypic variables with SNP allele frequencies after accounting for the genetic structure. We imputed the missing data and performed the LFMM analyses as described in our previous paper [51]. A list of candidate loci for each explanatory variable was then generated with an FDR of 1% and adjusted *p*-values of < 0.001. Genetic differentiation (*F*_*ST*_) between populations was calculated for each dataset of adaptive SNPs using VCFtools [106].

### Current and future predictions of adaptive genetic variation

A gradient forest (GF) approach was used to model the present and future patterns of genetic variation. It is a regression tree approach which allows for modeling the turnover in genetic composition to accommodate nonlinear associations of spatial, environmental, and allelic variables [12, 113]. Using GF methods as described in [12], we modeled climatic and spatial factors influencing genomic variation for five datasets of SNP: (a) the complete SNP set; (b) SNPs associated with geography; (c) the significant associated SNPs to climate; (d) SNPs associated with phenotypic traits (individual and family together); and (e)* F*_*ST*_ outliers. For each model for LFMM analysis, six climatic variables were selected. The method of Moran’s eigenvector maps (MEM), implemented in “spacemakeR” 0.0–5 package in R, was used to define spatial variables [117]. Half of the positive MEM variables were retained as has been suggested in similar studies [9, 29, 87]. Then, by running GF with 2000 regression trees per SNP, maxLevel = log_2_(0.368*n*)/2, and a variable correlation threshold of 0.5, the importance of conditional variables was determined as suggested [113, 114]. Other parameters were set to default values. All GF analyses were conducted in R, using the “gradientForest” 0.1–32 package [113].

To make the GF results visualization, principal component analysis (PCA) was used to transform the environmental variables into new synthetic variables. The first three principal components (PCs) were used to create a red–green–blue (RGB) color palette. A Procrustes superimposition [115] on the PCAs was utilized to compare mapped genetic composition for all SNP sets and four adaptive SNP sets as described in Martins et al. [29]. The Procrustes residuals represent the absolute distance in genetic composition between SNP datasets at each location.

In the end, the GF was used to determine the genetic offset under predicted future climate. The genetic offset is a predictive measure to identify the regions where the relationship between genes and the environment will be the most disrupted between the current and future climates [12]. The analyses were performed for the complete dataset of SNPs and the subsets of SNPs related to geography, climate, and phenotypic traits. To assess vulnerability to climate change, BioClim variables for future climate were obtained for the year 2080 under the GFDL-ESM4 model [116]. For every grid cell, the Euclidean distances between the present and future genetic compositions were computed and used as a measure of genetic offset [113].

### Supplementary Information

**Additional file 1.**

**Additional file 2.**

## Data Availability

Illumina data are publicly available at the NCBI BioProject PRJNA935374. (https://www.ncbi.nlm.nih.gov/bioproject/PRJNA935374). A dataset of 18,799 SNPs in VCF format is available on Zenodo: https://doi.org/10.5281/zenodo.7732733.
