# Supplementary material for: Signatures of local adaptation to current and future climate in phenology-related genes in natural populations of Quercus robur
Source: BMC Genomics. 2024 Jan 19;25:78. doi: 10.1186/s12864-023-09897-y (PMC10797717; doi:10.1186/s12864-023-09897-y)
Supplement: Supplementary file 2 — Additional file 2. [file 12864_2023_9897_MOESM2_ESM.docx]

**Supplementary Information**

**Additional file 2**

**Figure S1.** Results of the STRUCTURE analysis in *Quercus robur*. (A) The magnitude of ΔK as a function of *K*, with *K*-values ranging from 1 to 10. (B) Likelihood of *K* for each value of *K*.

**Figure S2.** Membership coefficients per individual at *K* = 4 clusters inferred from STRUCTURE.

**Figure S3.** Cumulative importance of allelic change along spatial (MEMs) and climate gradients for all SNPs and three adaptive SNP datasets.

| A  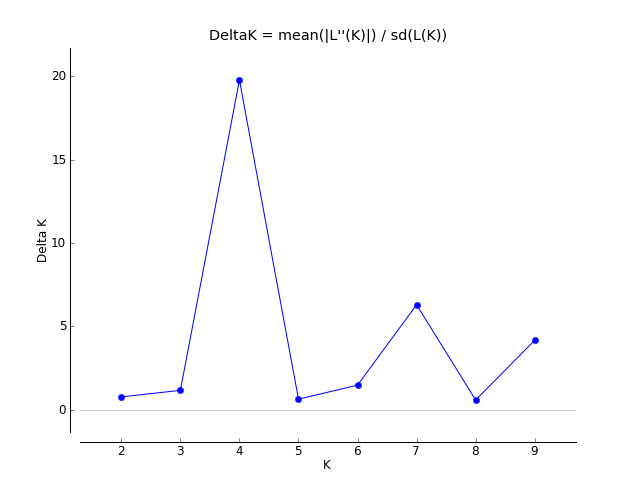 | B  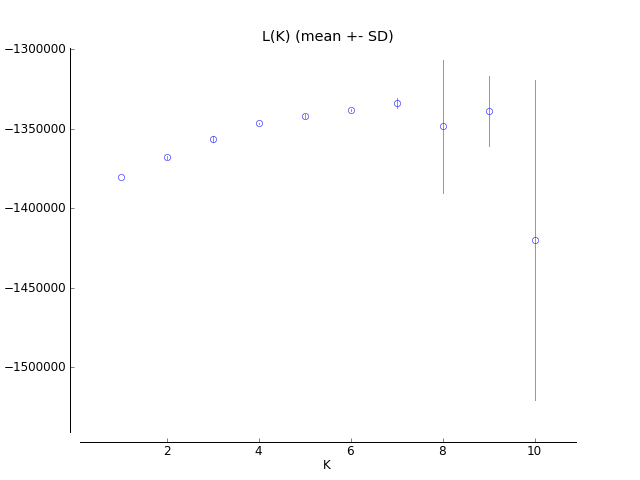 |
| --- | --- |

**Figure S1.** Results of the STRUCTURE analysis in *Quercus robur*. (A) Magnitude of ΔK as
a function of K, with K-values ranging from 1 to 10. (B) Likelihood of K for each value of K.


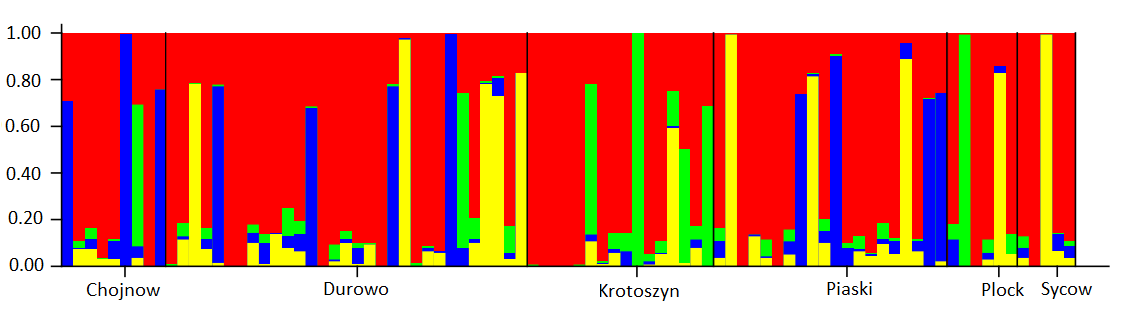


**Figure S2.** Membership coefficients per individual at K = 4 clusters inferred from STRUCTURE.

| 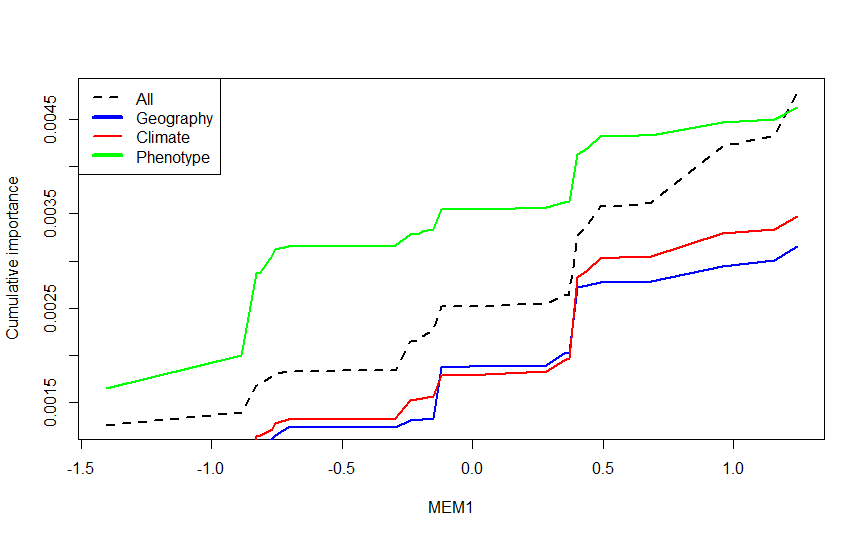 | 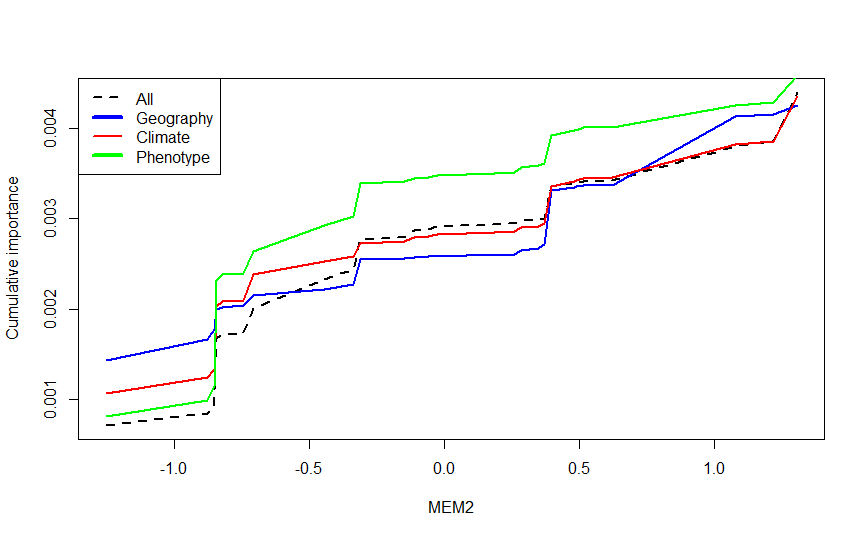 |
| --- | --- |
| 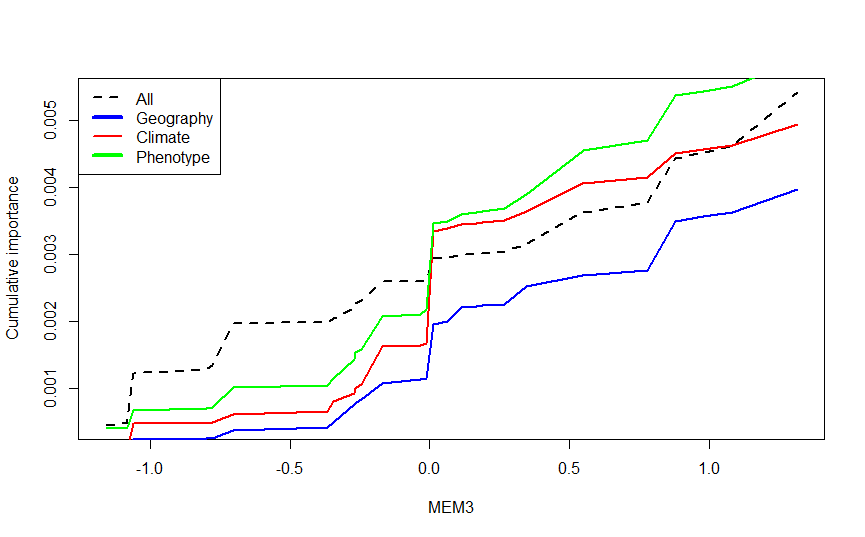 | 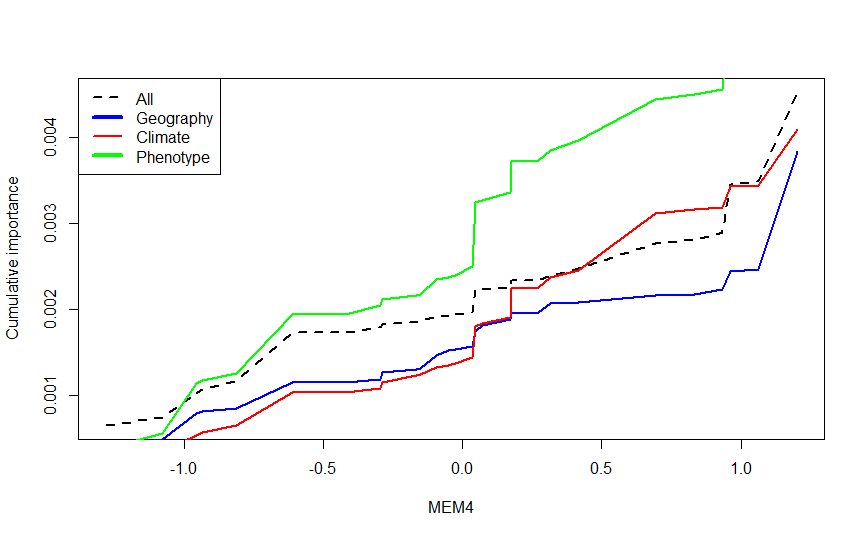 |
| 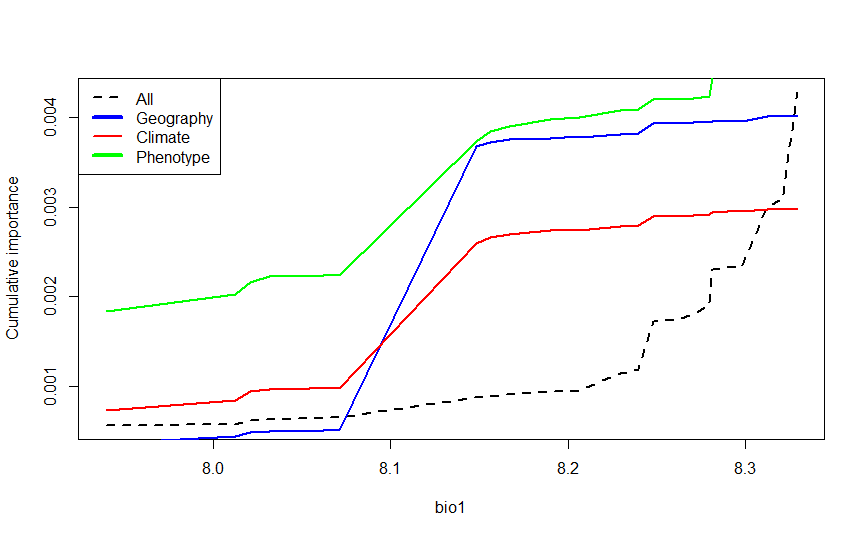 | 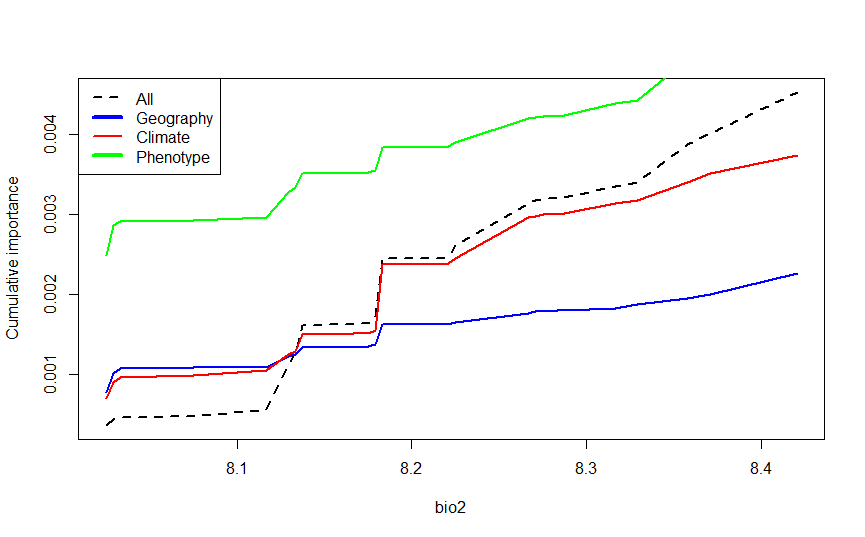 |
| 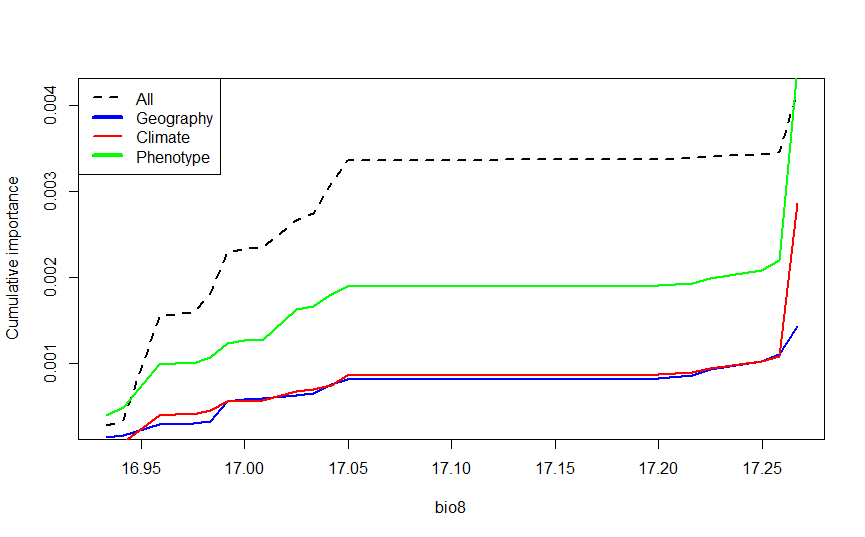 | 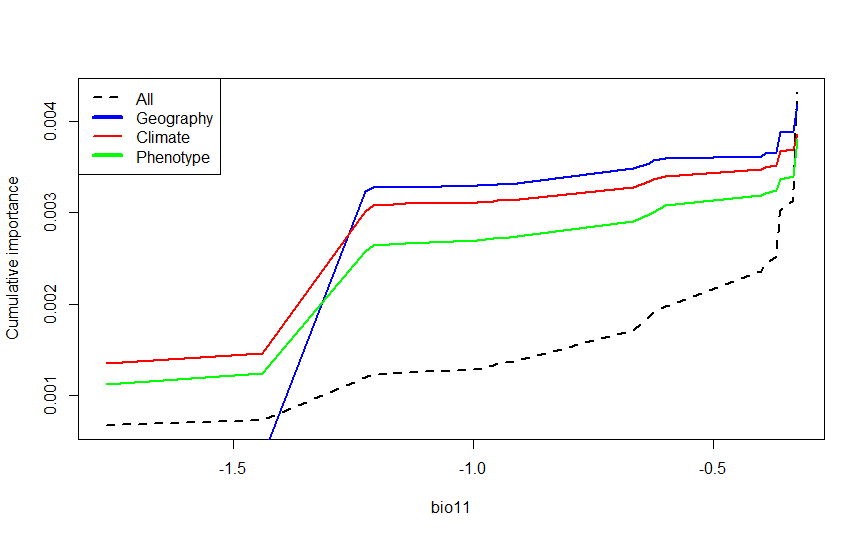 |
| 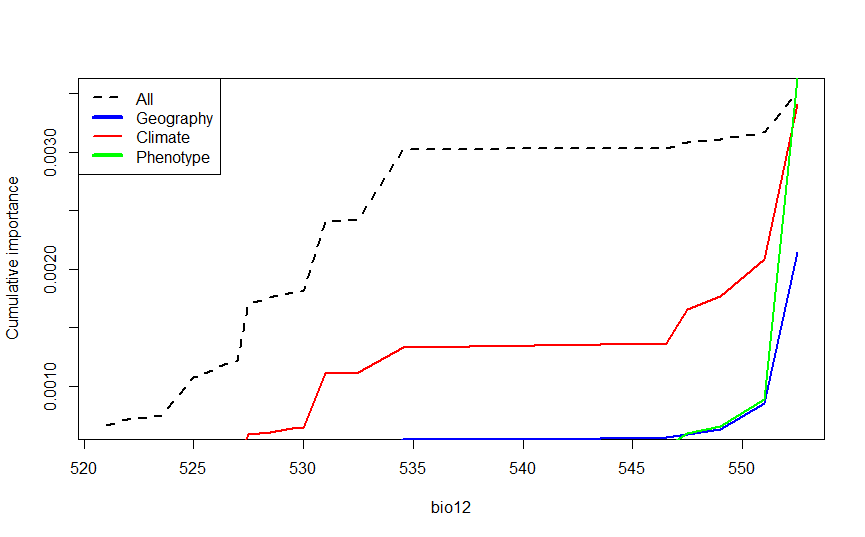 | 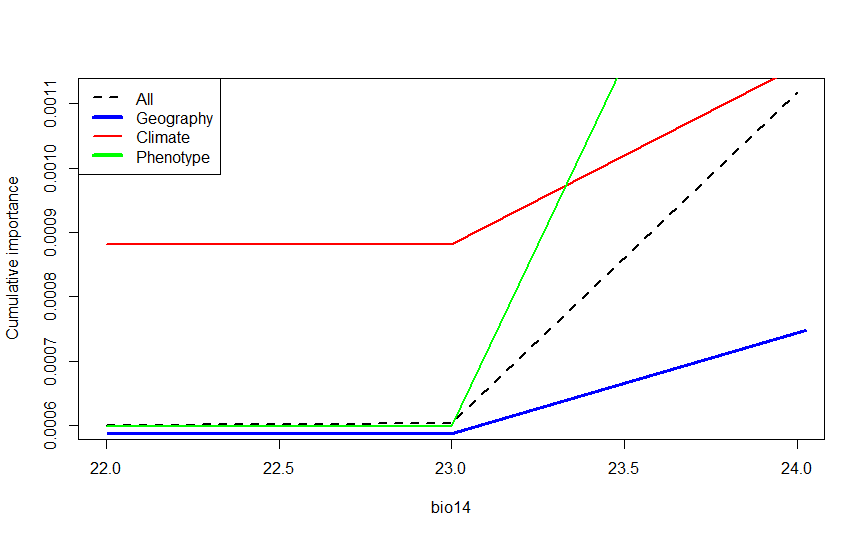 |

**Figure S3.** Cumulative importance of allelic change along spatial (MEMs) and environmental gradients for all SNPs and three adaptive SNP datasets.
